# PCAF acetylates AIB1 to form a transcriptional coactivator complex to promote glycolysis in endometrial cancer

**DOI:** 10.3389/fonc.2024.1442965

**Published:** 2024-09-05

**Authors:** Di Wu, Mingxia Li, Mingyang Wang, Zhifeng Yan, Yuanguang Meng

**Affiliations:** ^1^ School of Medicine, Nankai University, Tianjin, China; ^2^ Department of Obstetrics and Gynecology, The First Affiliated Center of Chinese People’s Liberation Army (PLA) General Hospital, Beijing, China; ^3^ Department of Obstetrics and Gynecology, The Seventh Medical Center of Chinese PLA General Hospital, Beijing, China

**Keywords:** endometrial cancer, aerobic glycolysis, amplified in breast cancer 1, P300/CBP-associated factor, molecular target

## Abstract

**Introduction:**

Despite rapid advances in molecular biology, personalized molecular therapy remains a clinical challenge for endometrial cancer due to its complex and heterogeneous tumor microenvironment.Based on clinical findings, AIB1 is a marker molecule for poor prognosis in endometrial cancer and may serve as a potential therapeutic target. Moreover, it is well known that aerobic glycolysis plays an important role in tumour energy metabolism. It has been previously reported in various hormone-related tumour studies that AIB1 affects glycolysis and promotes tumour development. However, the link between AIB1 and aerobic glycolysis in estrogen-dependent endometrial cancer remains unclear.

**Methods:**

We used two endometrial cancer cell lines to validate the high expression of target genes and the effect on the proliferative and invasive capacity of the tumours and verified the pattern of interactions and epigenetic modifications by CHIP and CO-IP techniques. Finally, the conclusions were validated on homozygous mice

**Results:**

In this study, we investigated the transcriptional co-activation functions of AIB1, including its acetylation by PCAF, binding to the c-myc transcription factor, and recruitment of glycolysis-related gene promoters.

**Discussion:**

Our findings provide new clues that perturbation of normal homeostatic levels of AIB1 is linked with endometrial cancer. These findings suggest that targeting AIB1-mediated regulation of aerobic glycolysis may offer a novel therapeutic approach for endometrial cancer with high AIB1 expression, opening new avenues for personalized diagnostics and treatment strategies in this disease.

## Graphical Abstract

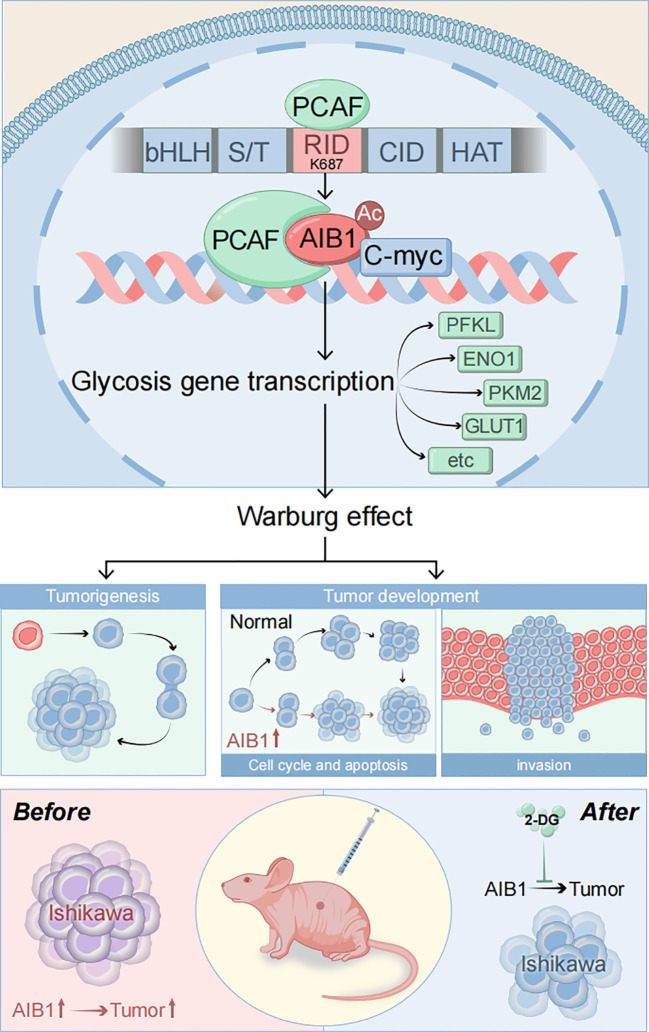

## 1 Introduction

Endometrial cancer is one of the most common gynecological malignancies worldwide, and it ranks sixth in incidence among female malignant tumors globally in global cancer statistics 2020 (1).Furthermore, 66,200 new cases of EC and 13,030 EC-related deaths in the US were estimated for 2023 (2). However, in emerging economies like China, the incidence of endometrial cancer has been rising significantly in recent years and trending toward a younger patient population (3). For example, a 2022 study found that the incidence of endometrial cancer in China has been increasing by approximately 3% annually. This calls for an active search for optimized cancer control strategies, especially in developing countries experiencing rapid social and economic changes. Early diagnosis and precise prognostic assessment are particularly effective ways to control endometrial cancer. In conjunction with current developments in molecular biology, individualized diagnosis and treatment based on molecular markers need to be integrated into existing health plans in order to cope with complex endometrial cancers that are increasingly exposed globally or for which treatment options are still limited.

Therefore, studying the mechanisms underlying endometrial cancer development and identifying key regulatory molecules are crucial for promoting early diagnosis, understanding individualized differences, and achieving precise treatment to improve prognosis. Energy metabolism disorder is an important mechanism for tumor occurrence and development. German biochemist Otto Warburg discovered that tumor cells primarily obtain energy through glycolysis, even under conditions of sufficient oxygen supply—a phenomenon known as “aerobic glycolysis” or the “Warburg effect”. (4).Glycolysis plays a vital role in various pathological processes associated with cancer (5). Active glycolysis improves the tolerance of tumor cells to ischemia and hypoxia conditions and avoids apoptosis caused by the inhibition of oxidative phosphorylation; secondly, tumor cells can utilize the intermediates of glycolysis or provide raw materials for anabolism through upregulation of the pentose phosphate pathway to satisfy the rapid proliferation of tumor cells. Thirdly, abnormal alterations in the function of key enzymes involved in gluconeogenesis can often lead to tumorigenesis. Additionally, the accumulation of lactic acid, the end product of aberrant glucose metabolism in tumors, can create an acidic local environment that disrupts the cellular matrix and promotes tumor invasion.

Emerging evidence indicates that the glycolytic processes of tumor cells, and the key regulatory factors, represent promising targets for cancer diagnosis and treatment. (6–8). Amplified in breast cancer 1 (AIB1), as a member of the steroid hormone receptor co activator family (SRC), interacts with multiple transcription factors to enhance their transcriptional activity. Studies have shown that the overexpression of AIB1 can affect various signaling pathways by promoting glycolysis, thereby initiating the development of diverse cancers, including breast, colorectal, and liver. (9–11).The posttranslational modification of AIB1, such as acetylation by the histone acetyltransferase PCAF, is crucial for regulating its protein expression and activity. PCAF can acetylate non-histone substrates, including AIB1, p53, and NF-κB, and participate in various cellular processes like proliferation, differentiation, apoptosis, and DNA damage repair. (12–14).

However, the interaction site of PCAF and AIB1 and the molecular mechanism of whether they can affect tumorigenesis and development through regulation of glycolytic metabolism remain unclear. Therefore, this study aims to elucidate how acetylation of AIB1 by PCAF promotes aerobic glycolysis and proliferation in endometrial cancer.

## 2 Materials and methods

### 2.1 Cell lines and cell culture

HEC-1A and Ishikawa cells were cultured in DMEM. The HEC-1A cell was obtained from the Academy of Military Medical Sciences and was free of mycoplasma contamination. Ishikawa was obtained from Cellverse Company (article number: iCell-h113) in Shanghai. Cell lines were validated using the short tandem repeat (STR) method. All media were supplemented with 10% fetal bovine serum and 1% penicillin–streptomycin. All cells were cultured at 37°C in an atmosphere containing 5% CO_2_ and 70%–80% humidity.

### 2.2 Clinical samples

A total of 112 patients suffering from endometrial cancer who have accepted standard surgery were obtained from the First Affiliated Hospital of PLA General Hospital prior to the study. All of the samples were embedded in paraffin. These patients did not undergo any therapeutic intervention before surgery. Two senior pathologists from the hospital’s pathology department examined all pathological tissue in accordance with World Health Organization standards.

### 2.3 siRNAs and plasmids

Oligonucleotides of siRNA targeting NCOA3 and PCAF respectively, and control siRNA were produced by JTS Scientific (Wuhan, China). The sequences are presented in **Supplementary Table 1**. Cells were transfected with the siRNAs at a final siRNA concentration of 50 nM using Lipofectamine 2000 (Invitrogen), according to the manufacturer’s instructions. The human pcDNA3.1-Myc-NCOA3 plasmid was purchased from Gene-bio (Henan, China). The human pcDNA3.1-Flag-PCAF plasmid was a gift from Professor Xiaojie Xu at Academy of Military Medical Sciences. DNA sequencing and enzyme digestion identification were performed to confirm plasmids integrity and accuracy.

### 2.4 Construction of stable knockdown and overexpression cell lines

Firstly, overexpression and knockdown constructs were generated using the lentiviral backbone vector pLVX-CMV-puro and the shRNA vector SHC201-u6-puro, respectively. Secondly, pspax2 and pMD2G were selected as auxiliary plasmids to form the lentiviral packaging system. Thirdly, 293t adherent cells were prepared with a convergence rate of 70% and the plasmids transfected in a 4:3:1 ratio. After 72 h, the lentiviral supernatant was collected, the viral titer was determined by fluorescence microscopy, and the virus was stored at −80°C for future use. Finally, the Ishikawa and HEC-1A cell lines were infected with the lentivirus, and stable knockdown cell lines were selected using puromycin.

### 2.5 RNA extraction and quantitative reverse transcription polymerase chain reaction

The TRIzol reagent (Invitrogen) was used to extract total RNA from cells in accordance with manufacturer instructions, and PrimeScript RT reagent kit (Vazyme, Nanjing, China) was used to transform RNA to cDNA. Quantitative RT-PCR (qRT-PCR) was performed on a CFX96 Dice™ real-time PCR system (Bio-Rad Laboratories, Inc., CA) using Taq Pro Universal SYBR qPCR Master Mix (Vazyme, Nanjing, China). Each sample was run in triplicates. The sequence of primers used in the study are presented in **Supplementary Table 2**.

### 2.6 Western blotting

Proteins were extracted from cells with the addition of protease inhibitors to prevent degradation during the lysis process. Protein concentrations were then quantified using the BCA method to ensure accurate results. Target proteins were separated via SDS-PAGE electrophoresis, followed by incubation with primary and secondary antibodies. The specific protein antibodies involved in this study include DYKDDDDK Tag Monoclonal Antibody (FG4R) (Invitrogen, MA1-91878); Myc Tag Antibody (PA1-981); c-Myc Antibody (MA1-980); PCAF Antibody (703379); and AIB1 Antibody (MA5-15898).

### 2.7 Cell proliferation

The CCK-8 and colony formation assays were utilized to assess cell viability and proliferation. For the CCK-8 assay, cells were seeded in six-well plates and incubated with the experimental conditions. After the desired timepoint, the CCK-8 reagent was added and the absorbance at 450 nm was measured using a spectrophotometer, which correlates with the number of viable cells. For the colony formation assay, cells were seeded, treated with experimental conditions, and then incubated to allow colony formation. The colonies were then fixed, stained with methylene blue, and quantified.

### 2.8 Cell cycle and apoptosis

Cells were synchronized, fixed, and permeabilized to preserve cellular structures and DNA integrity. The cells were then stained with propidium iodide (PI) and subjected to flow cytometry analysis to assess cell cycle distribution. Annexin V staining was also performed to detect apoptosis.

### 2.9 Migration and invasion assays

For the Transwell migration assay, cells are seeded in the top chamber of a Transwell insert with a porous membrane, whereas the bottom chamber contained serum-containing medium. After incubation, the cells on the top or bottom of the membrane were fixed, stained, and quantified using image analysis. The invasion assay was similar, but the membrane was coated with Matrigel to assess the cells’ invasive capacity.

### 2.10 Coimmunoprecipitation assay

Starting by lysing the cells to obtain the supernatant, the indicated antibody was added for incubation overnight with shaking at 4°C. The next morning, washed Protein A/G magnetic beads were added to the antigen–antibody complex system for continued incubation. Sediment was centrifuged and retained. After washing three times with IP washing buffer, loading buffer was added to the complex system and denatured at 100°C for 10 min. Then, the samples were subjected to immunoblot analysis.

### 2.11 Chromatin immunoprecipitation

The first step was to fix 2 × 10^6^ cells/ml with 1% formaldehyde for 10 min at room temperature. Glycine (0.125 M) was added immediately to quench for 8 min at 37°C and wash three times with PBS. Next, the nuclei were extracted after ultrasonic disruption. The third step was to incubate overnight at 4°C with an anti-c-Myc antibody or IgG, along with Protein A/G magnetic beads. Afterward, washing complexes with high-salt solutions, purified DNA was obtained for subsequent quantitative PCR (qPCR) analysis. The primer sequences for the glucose metabolism related gene promoter are provided in **Supplementary Table**.

### 2.12 Immunoblotting

Endometrial cancer tissue chips containing 37 patients were purchased from Ximin Trading company (Qingdao, China), and Jiankun Herun Technology Company (Beijing, China) was commissioned to provide immunohistochemical testing services.

### 2.13 Cellular energy metabolism assays

Mammalian cells have two key energy metabolism pathways, aerobic respiration and glycolysis. Aerobic respiration takes place in the cytoplasm and mitochondria, and the process of consuming oxygen drives the cell to oxidize and break down the nutrient substrates (sugars, lipids, proteins) and release energy to synthesize large amounts of ATP, so the mitochondria are also known as the “energy factory” of the cell. Glycolysis occurs in the cytoplasm of cells and is an anaerobic decomposition process that mainly breaks down glucose into lactic acid and produces small amounts of ATP. Glycolysis and oxidative phosphorylation are two key energy-producing pathways in cells. Most cells have the ability to switch between these two pathways, adapting to changes in their environment.

Extracellular acidification rate (ECAR) and oxygen consumption rate (OCR) were measured with a XFe96 Analyzer. Briefly, cells digested to a density of 1 × 104/well were seeded in XFe96 assay plates (Agilent Technologies, Santa Clara, CA, USA) and were then placed in an incubator of 37°C and 5% CO_2_ for 24 h. Simultaneously, an XFe96 cartridge was hydrated the day prior to the XF assay. Then, XF Real Time ATP Rate Assay Medium was prepared and warmed to 37°C at the day of assay. Next, the cell culture plate was retrieved from the CO_2_ incubator and the cells’ state was viewed. Around 1 h before detection, culture medium was replaced by XF Real-Time ATP Rate Assay Medium (Agilent Technologies). Subsequently, the cells were treated sequentially with 1 μM of glucose, 1 μM of oligomycin (ATP synthase inhibitor), and 0.5 μM of 2-DG (the glycolytic inhibitor) at time points for measurement of ECAR. For OCR, once all required ports were filled with drugs, the cartridge and utility plate were transferred to the XFe96/XF96 instrument and cartridge calibration was started using the assay template created before.

### 2.14 Animal studies

We purchased 30 BALB/c nude mice (female, 4–5 weeks old, 16–18 g in body weight) from Vital River Company (Beijing, China) and randomly assigned them to six groups (n = 5 per group). Each group of mice was placed in a cage for feeding. The mice were housed at the specific pathogen-free (SPF) facility following the principles of animal welfare strictly. The animal experiment was divided into six groups: the control group compared with overexpression, the AIB1 overexpression group, the AIB1 overexpression and administration of 2-DG group, the control group compared with knockdown, the AIB1 knockdown group, and the AIB1 knockdown and administration of 2-DG group. The administration method of 2-DG is intraperitoneal injection of 500 mg/kg. A tumor-bearing mouse model was established by subcutaneously injecting 100 μl of the transfected cells into the mice. Tumor volume was measured using a vernier caliper every 4 days and quantified using the following formula: Volume (mm^3^)=length×width^2^/2. After 28 days’ measurement or humane end point, the mice were sacrificed. Subsequently, the tumors were isolated from all mice.

### 2.15 Bioinformatics analysis

We retrieved expression data and clinical information from the Department of Obstetrics and Gynecology of the First Medical Center of the General Hospital of the Chinese People’s Liberation Army. The LIMMA package was used to identify DEGs between good and poor prognosis endometrial cancer tissue samples. An adjusted P < 0.05 and an absolute log2 FC > 1 were considered statistically significant. To determine the potential biological processes and pathways of the overlapping DEGs, ingenuity pathway analysis (IPA, www.qiagen.com/ingenuity) (accessed on 03 July 2020) and protein interaction network was performed, with P < 0.01 and absolute log2FC > 1 as the threshold values.

### 2.16 Statistical analyses

GraphPad Prism 8.4.2 was used for data analysis. Data were expressed as mean ± SD. Comparisons between two groups were performed by T-test, comparisons among multiple groups were performed by one-way ANOVA. P-values < 0.05 indicate statistical significance. * represents 0.01<P ≤ 0.05, ** represents 0.001<P ≤ 0.01, *** represents P<0.001.

## 3 Results

### 3.1 AIB1 is a novel oncogene and associated with poor prognosis

**Figure 1:**
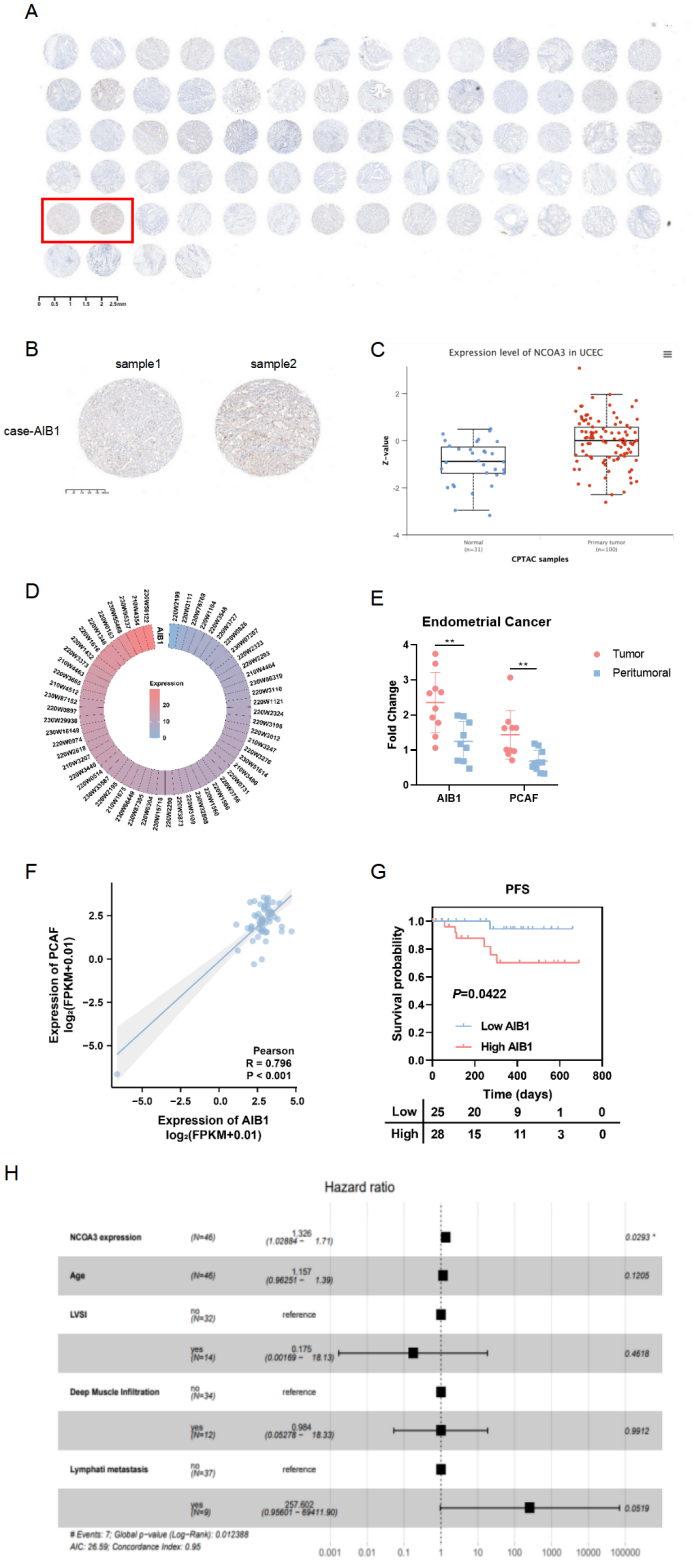
AIB1 is enriched and predicts outcome in endometrial cancer. **(A)** Tumor tissue microarrays containing 74 loci from 37 endometrial cancer patients. Also, immunohistochemistry **(IHC)** protein analysis of AIB1 was completed in the above samples. **(B)** Results of applying IHC to detect AIB1 expression at different sites of tumor tissues of the same patient. **(C)** High AIB1 expression is associated with poor prognosis in tumor patients according to CPTAC database information. **(D)** Heat map of AIB1 expression in clinical endometrial cancer patients detected by application of second-generation gene sequencing technology. **(E)** There were 10 endometrial cancer patients with good concordance selected to compare the expression of AIB1 and PCAF in tumor and paracancerous tissues using RT-PCR assays. **(F)** Positive correlation between PCAF and AIB1 expression based on genetic testing of clinical patients. **(G)** Kaplan–Meier analysis of progression-free survival (PFS) according to mRNA expression of AIB1 in clinical patients (n = 112). Blue dots represent patients with low AIB1 expression, and red dots represent patient with high AIB1 expression. **(H)** Multifactorial survival analysis of 112 endometrial cancer tissues from clinical samples using PFS as the primary outcome indicator. High AIB1 expression is an independent prognostic influencer in endometrial cancer patients (P=0.0293). (*P<.05, **P<.01, ***P<.001).

Consistent with the Clinical Proteomic Tumor Analysis Consortium (CPTAC) analysis (**Figure 1C**), tissue microarray (Ximin Trading Company) results from 37 patients with endometrial cancer showed that AIB1 was highly expressed in tumor tissue, as well as in the nucleoplasm and cytoplasm (**Figures 1A, B**). AIB1 gene expression was detected correspondingly in 57 clinical samples sourced from the General Hospital of the People’s Liberation Army (PLA), as shown in **Figure 1D**. Tissue samples from 10 of these patients were randomly selected and tested for central and paracancerous AIB1 and PCAF expression. It was found that both molecules were significantly more highly expressed in the tumor compared with the paraneoplastic tissue, and the difference was statistically significant (P<0.01) (**Figure 1E**). Moreover, a positive correlation was found between AIB1 and PCAF expression in **Figure 1F** (R=0.796, P<0.001). Subsequently, long-term follow-up of 112 endometrial cancer patients with complete genomic and transcriptomic molecular information in our institution showed that high AIB1 expression predicted poor prognosis (P=0.042)(**Figure 1G**). In addition, high AIB1 expression was also confirmed to be an independent risk factor for shorter progression-free survival by a multifactorial prognostic analysis (P=0.029)(**Figure 1H**).

### 3.2 AIB1 promotes tumor proliferation and invasion

**Figure 2:**
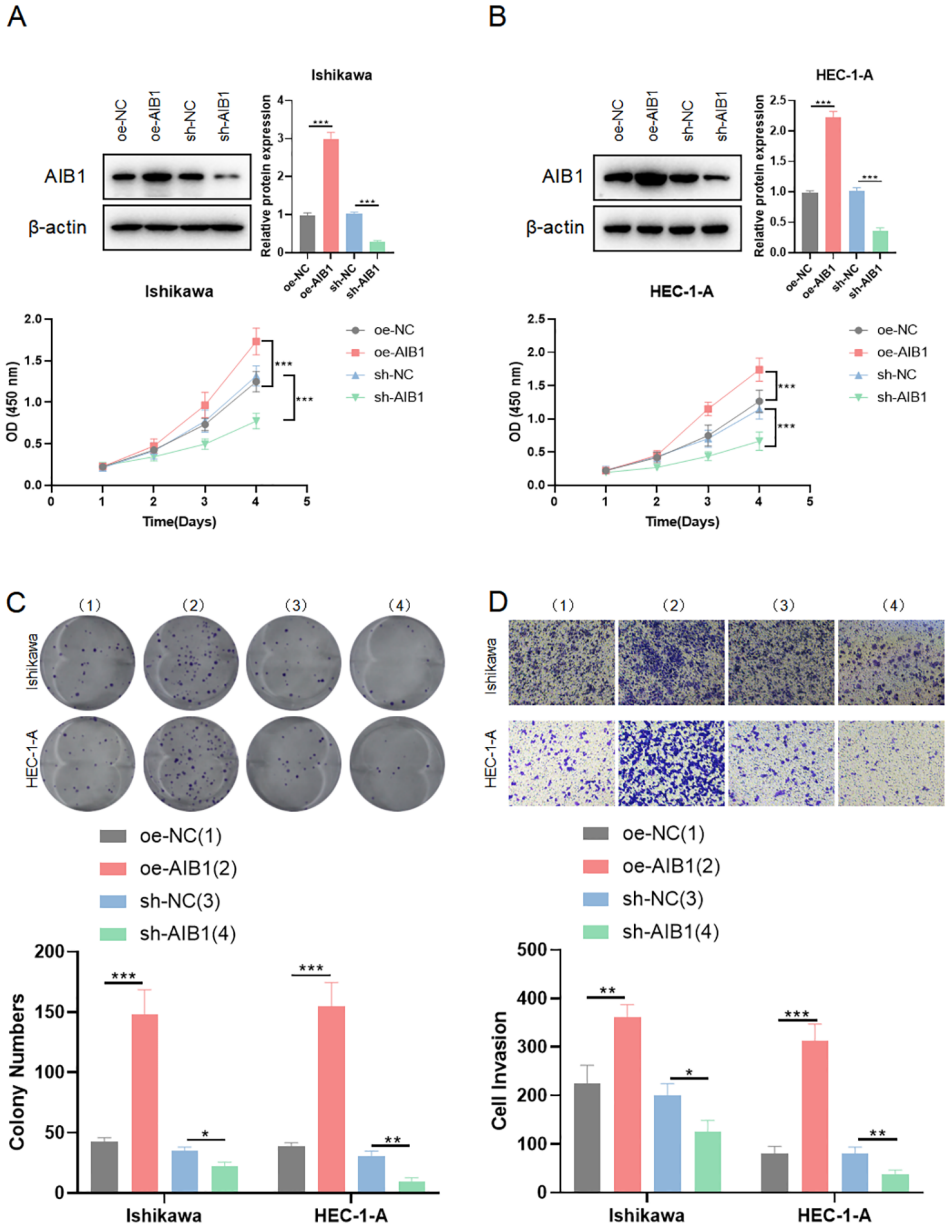
AIB1 affects endometrial cancer cell proliferation and infiltration. **(A, B)** Cells were transfected with either sh-NC and oe-NC or sh-AIB1 and oe-AIB1. Cell proliferation was measured after 5 days. Graphs display absorbance measured at 450 nm using an enzyme meter in Ishikawa **(A)** and HEC-1-A **(B)**. **(C)** Colony forming assay was carried out with cells treated with either sh-NC and oe-NC or sh-AIB1 and oe-AIB1 for a total of 14 days. Representative images of the cells stained with crystal violet at day 14. Graph displays colony numbers in different conditions in two cell lines. All experiments are representative of three biologically independent replicates. Two-sided t-tests were used to calculate P values (***P ≤0.001,**P ≤ 0.01,*P ≤ 0.05). **(D)** Transwell assay was performed to assess the migration ability of Ishikawa and HEC-1-A cells. The grouping and replications were the same as in the colony forming assay.

To investigate the impact of AIB1 on EC cell line growth and proliferation, AIB1 overexpression and knockdown efficiency were confirmed by immunoblotting experiments. Next, the effects of AIB1 overexpression and knockdown status on cell proliferation viability were verified using two cell lines, HEC-1A and Ishikawa, respectively. The results showed that AIB1 overexpression significantly increased tumor proliferation capacity. Conversely, the effect was significantly weakened in knockdown (**Figures 2A, B**). Then, plate cloning experiments again verified the proto-oncogene function of AIB1, confirming that tumor cells highly express the gene and promote tumor growth (P<0.001). Conversely, knocking down the gene inhibits tumor growth (P<0.05) (**Figure 2C**). In addition, the results of the invasion assay showed that overexpression of AIB1 significantly enhanced the migratory infiltration ability of tumor cells (P<0.01) and vice versa (P<0.05) (**Figure 2D**). The differences were all statistically significant.

### 3.3 AIB1 affects cell cycle and programmed death processes

**Figure 3:**
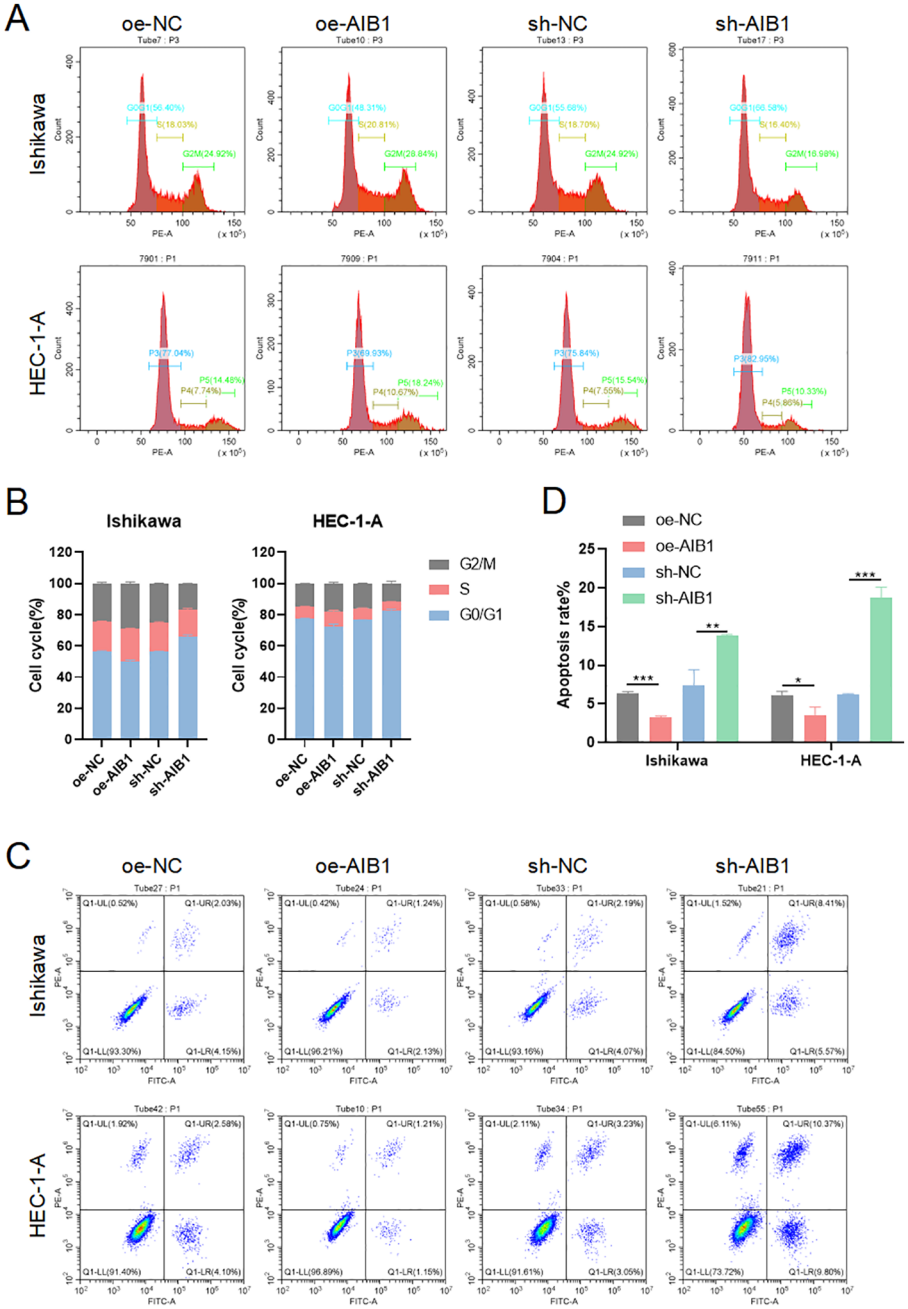
AIB1 Induces cell cycle and disrupts apoptosis. **(A)** The effect of AIB1 knockdown and overexpression on the EC cell cycle. **(B)** AIB1 knockdown increased the proportion of cells in the G1 phase in both EC cell lines. (*P < .05, **P < .01, ***P < .001). **(C)** Endometrial cancer cell apoptosis was detected by flow cytometry analysis. **(D)** The graph displays the apoptosis rate of both Ishikawa and HEC-1-A cell lines. Compared with the NC control group, cells treated with the AIB1 knockdown apoptotic index were significantly higher. In opposite, high AIB1 expression disrupts regular programmed cell death.

The cell cycle is a highly regulated process that controls the growth and division of cells at the appropriate times and in the correct manner. It is divided into distinct phases to perform a series of events, each with specific functions and checkpoints to ensure accurate replication and division (15). Cells in G1 phase grow in size, synthesize proteins, and prepare for DNA replication. Immediately following the replication of the cell’s genetic material occurs in the S phase. The G2 phase is a period of growth and preparation for cell division (16). The mitosis phase encompasses the process of dividing the duplicated DNA and cellular contents into two daughter cells. To investigate whether growth inhibition upon AIB1 depletion is related to alterations in the cell-cycle profile of EC cells, we analyzed cellular DNA content with flow cytometry. As shown in **Figures 3A, B**, AIB1 knockdown in Ishikawa and HEC-1A cells induced G1 arrest. Overexpression of the proto-oncogene AIB1 affects cell cycle regulation, thereby disrupting the normal regulation of cell growth and division, leading to uncontrolled cell division and cancer development. Consistently, we also observed that the percentage of cells in the G1 phase decreased concurrently with an increased percentage in the S phase.

Apoptosis, the best-known form of programmed cell death, is a key physiological mechanism for limiting the expansion of cell populations, both to maintain tissue homeostasis and to remove potentially deleterious cells, such as those with persistent DNA damage (17). Loss of apoptosis can allow the survival and accumulation of abnormal cells, promoting tumor initiation. Impaired apoptosis can also contribute to tumor progression and metastasis. Cancer cells often acquire resistance to apoptosis, which enables them to evade cell death signals and survive in adverse conditions. The results of the present study show that AIB1-overexpressing endometrial cancer cell lines disrupt a regulated and evolutionarily conserved cell death program. However, when knocking down the AIB1 gene, cells activate apoptosis as an important tumor suppression strategy (**Figures 3C, D**).

### 3.4 AIB1 influences tumor occurrence and development by glycolysis

**Figure 4:**
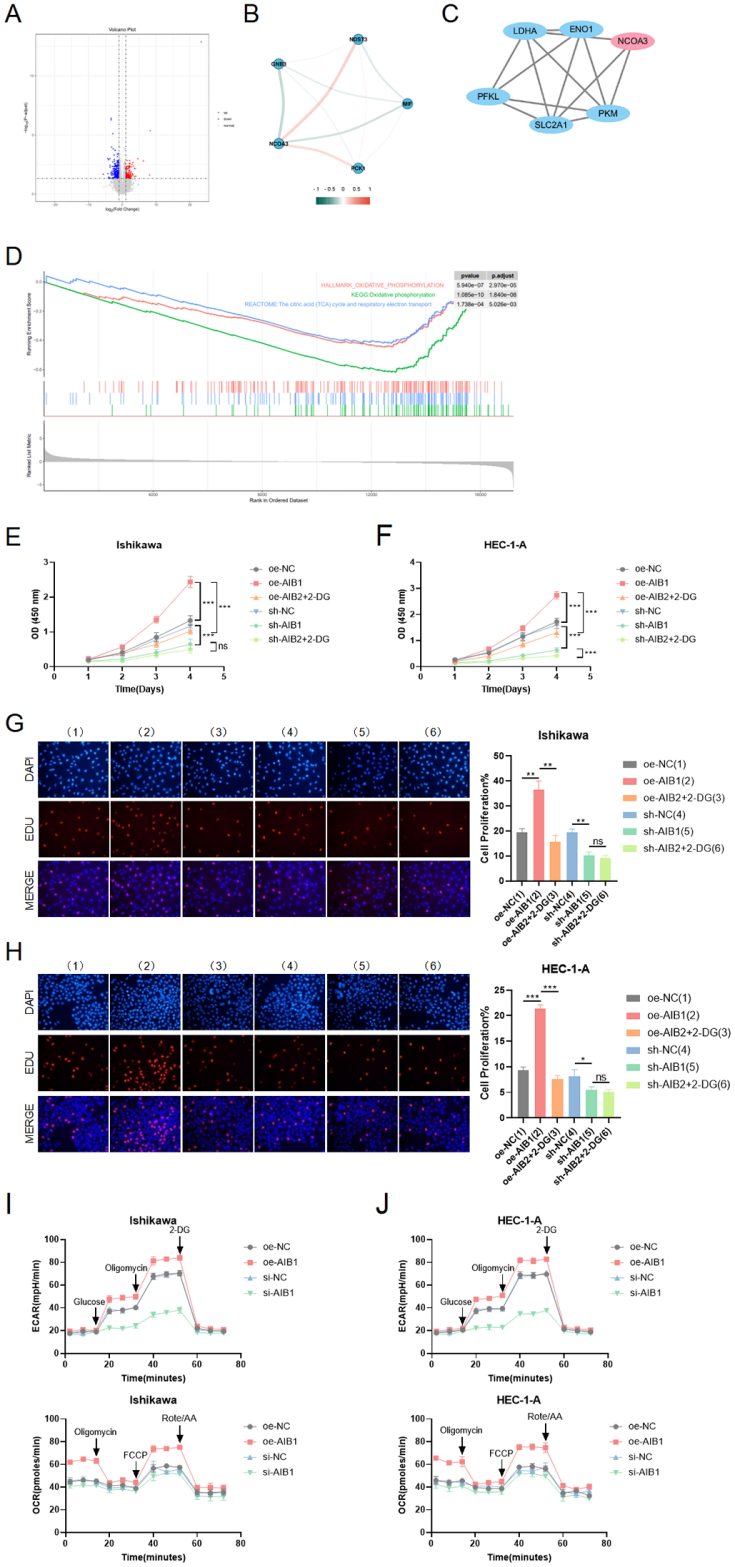
Role of AIB1 in tumor glucose metabolism. **(A)** Transcriptomics data from 112 clinical endometrial cancer patients were grouped according to AIB1 expression level, and volcano plots were drawn to compare the two groups of differential genes. Red dots and blue dots represent differential expression ((absolute log2 fold change >1 and adjusted P-value < 0.05), and gray sections are non-significant differential expression. **(B)** The graph shows the interrelationship of differential genes with AIB1 in the volcano map. **(C)** In protein−protein interaction (PPI) network analysis, it was found that AIB1 can directly or indirectly interact with several other glucose metabolism-related genes. Larger nodes represent stronger gene connectivity, and thicker lines indicate more reliable interconnections between genes and higher combined score values. **(D)** GSEA enrichment analysis showed that DEGs were significantly enriched in two glucose metabolism-related pathways, namely, oxidative phosphorylation and the citric acid (TCA) cycle and respiratory electron transport. These two pathways were significantly downregulated in the AIB1 high-expression group compared with the AIB1 low-expression group. In addition, differentially expressed genes were also significantly enriched in pathways mediating cell proliferation, such as the mTOR signaling pathway (KEGG enrichment analysis), the PI3K-Akt signaling pathway (GSEA enrichment analysis), and the PI3K-Akt signaling pathway (GSEA enrichment analysis). **(E, F)** The graphs demonstrate the effect on cell proliferation viability after addition of the glycolysis inhibitor (2-DG). The results were validated simultaneously in two cell lines, respectively. **(G, H)** EdU staining was used to show the effect of 2-DG drugs on cell proliferation in different treatment groups in two cell lines. The results further evaluate that the AIB1 gene affects tumors through glycolysis processes. **(I, J)** The extracellular acidification rate analyses of Ishikawa and HEC-1-A cells stably expressing knockdown control, shAIB1, 2-DG adding to shAIB1 and overexpression control, oeAIB1, 2-DG adding to oeAIB1. The oxygen consumption rate analysis as the above. ns P>.05, *P<.05, **P<.01, ***P<.001.

Given the crucial role of energy metabolism in cancer, we investigated whether AIB1 could influence tumorigenesis and progression through its impact on glycolysis, a key metabolic pathway in cancer cells. We previously successfully identified AIB1 as a prognostic differentially expressed gene using second-generation sequencing based on a grouping of endometrial cancer clinical samples with follow-up prognosis. Next, we analyzed the two groups of differentially expressed genes to form a volcano map based on the level of AIB1 expression (**Figure 4A**). Meanwhile, genes involved in glucose metabolism were screened to be closely related to AIB1 (**Figure 4B**). Moreover, the key proteins in the glycolysis process related to AIB1 were associated through a PPI network (**Figure 4C**). Immediately afterward, the differential gene enrichment analysis revealed that the high expression of AIB1 was associated with glucose metabolism and cell proliferation pathway (**Figure 4D**).

To determine the functional relevance of AIB1-mediated glycolysis, we performed rescue experiments by inhibiting glycolysis using a specific inhibitor, such as 2-deoxyglucose (2-DG). We found that the inhibition of glycolysis significantly attenuated the proliferative advantage conferred by AIB1 overexpression in two EC cell lines (P < 0.001), suggesting that AIB1 promotes tumorigenesis and progression, at least in part, through enhanced glycolytic metabolism. In **Figures 4E, F**, it was shown that 2-DG can significantly reverse AIB1 promotion of abnormal cell proliferation. Correspondingly, knockdown of AIB1 significantly attenuated the effect of glycolysis on cell viability. The results of the EDU assay were consistent between the two cell lines (**Figures 4G, H**).In addition, the results of the extracellular acidification assay (ECAR) and oxidative phosphorylation assay (OCR) confirmed that high expression of AIB1 promotes tumor glycolysis (**Figures 4I, J**).

### 3.5 PCAF acetylates AIB1 at K687 and binds as a transcriptional coactivator complex

**Figure 5:**
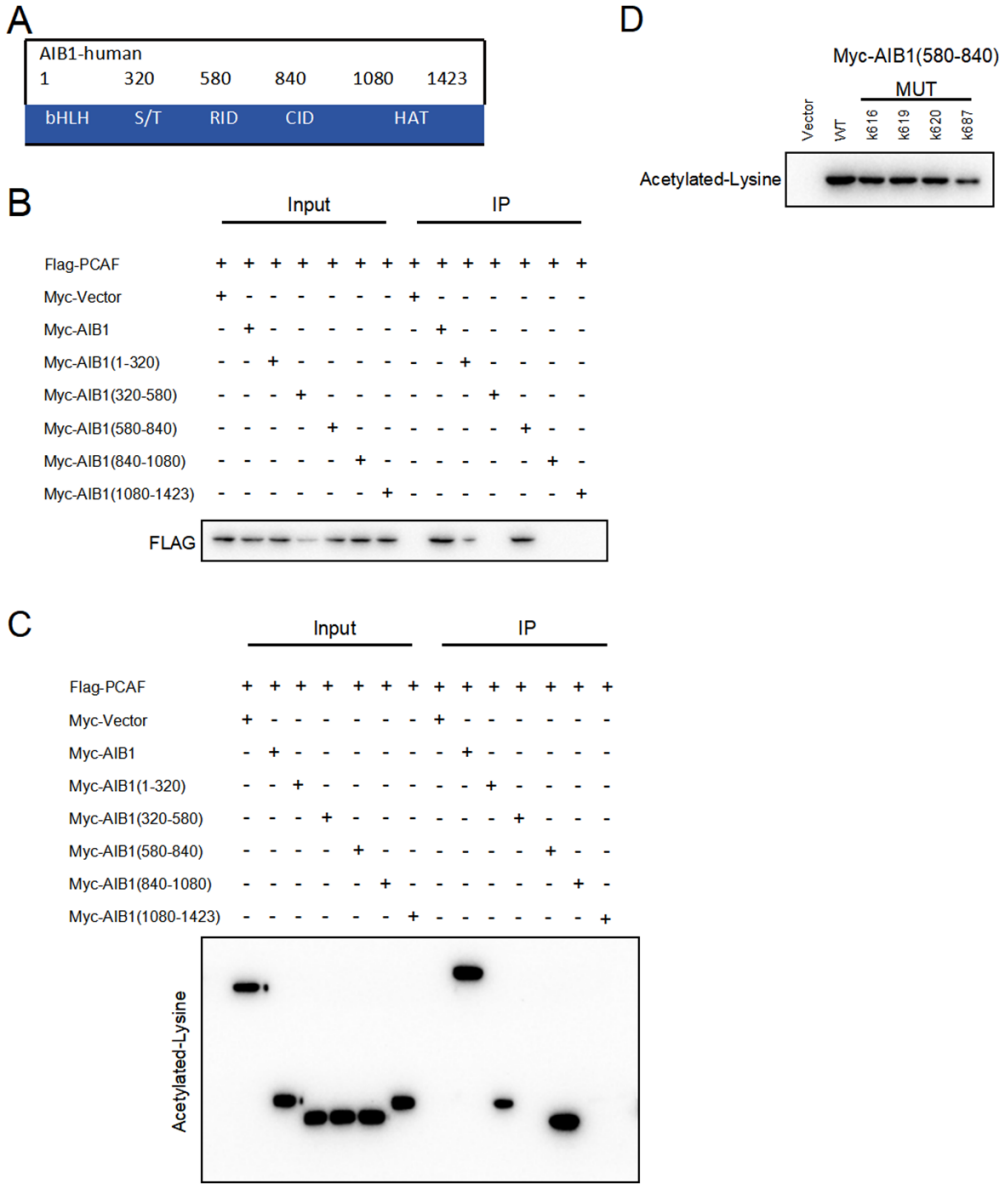
PCAF and AIB1 interact and acetylate at K687. **(A)** The AIBI protein consists of several functional structural domains, including the N-terminal bHLH, the serine/threonine-rich region, and C-terminal transcriptionally active structural domains. **(B)** Co-IP analysis of the interaction of Myc-AIB1 and Flag-PCA in Ishikawa cells. Flag antibody expression is detected by pulling with Myc-tagged magnetic beads. Results indicate that AIB1 interacts with PCAF through its RID domain (580 aa–840 aa). **(C)** PCAF acetylates the RID functional structural domain of AIB1. The interaction of AIB1 with PCAF was confirmed by the Co-IP assay. **(D)** PCAF acetylation AIB1 at k687.

AIB1 (amplified in breast cancer 1), also known as SRC-3 (steroid receptor coactivator 3), is a transcriptional coactivator protein that plays a crucial role in regulating gene expression. It consists of several distinct structural components including the following. N-terminal basic helix–loop–helix–Per/ARNT/Sim (bHLH–PAS) domain involved in DNA binding and heterodimerization between proteins containing these motifs is the most conserved region among SRC family members (18). The nuclear receptor interacting domain (RID) immediately following the serine/threonine-rich region (S/T) contains the LXXLL (where L is leucine and X is any amino acid) motif that is important for nuclear receptor binding. The intrinsic transcriptional activation domain (AD), which is responsible for interacting with the general transcriptional coactivator CBP/p300, is located at the c-terminus of the SRC molecule receptor interaction domain (19). Furthermore, the AIB1 C-terminal HAT structural domain may be involved in chromatin remodeling and assembly of the peripromoter transcription machinery during nuclear receptor-directed transcription initiation. The five functional domains of AIB1 are shown in **Figure 5A**. However, its importance in AIB1 transcriptional activation when considering activity remains to be clarified.

Previous studies have shown that posttranslational modifications of the AIB1 protein are pivotal in the regulation of gene processes (20).Our studies revealed that PCAF co-precipitated with AIB1 in extracts prepared in Ishikawa and HEC-1A cells and that overexpression of PCAF and AIB1 led to acetylation of AIB1 and formation of transcriptional co-activation complexes, which enhanced its transcriptional activity. In order to clarify the specific binding sites, we constructed plasmids with five separate structural domains of AIB1 with a Myc tag and full-length plasmids, respectively, and co-transfected them with Flag-PCAF plasmid in endometrial cancer cell lines, which not only proved the protein interactions but also identified the specific roles of PCAF and AIB1 in the region of amino acids 580–840 by immunoprecipitation (**Figure 5B**).

Next, based on the fact that PCAF has an acetyltransferase role, we further determined whether PCAF and AIB1 protein interactions are achieved through an acetylated form. We performed co-immunoprecipitation assay and acetylation antibody to confirm that PCAF greatly catalyzes the acetylation of AIB1 (especially in the 580–840 amino acid region) in endometrial cancer cells (**Figure 5C**). To further validate the acetylation active site of AIB1, we mutated the lysines at positions 616, 619, 620, and 687 to arginine to mimic deacetylation, resulting in an AIB1 acetylase inactivation mutant.

We subsequently found that the acetylation ability of K616, K619, and K620 mutants was similar to that of wild-type AIB1, but the acetylation level of K687 was significantly attenuated in the precipitates of PCAF and AIB1 interaction. These results suggest that PCAF acetylates AIB1 at K687 (**Figure 5D**).

### 3.6 Co-activation complex regulate downstream glycolysis through c-myc

**Figure 6:**
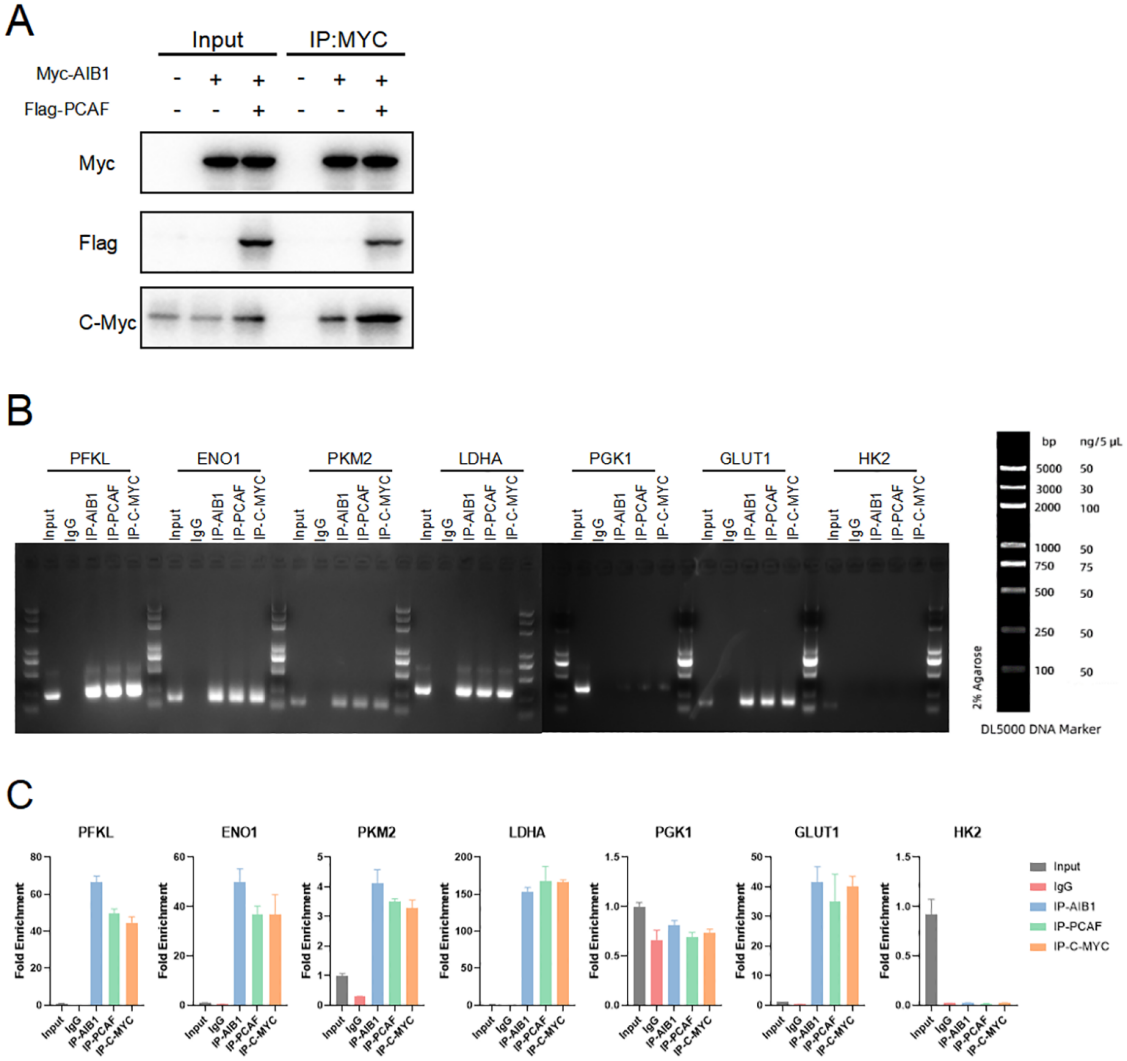
Glycolysis gene expression is regulated by AIB1 and PCAF complex by mediating c-myc transcriptional activity. **(A)** COIP analysis showed that both PCAF and AIB1 could bind to native c-myc alone, but complex formation bound c-myc more pronouncedly. **(B, C)** ChIP analysis of the AIB1 complex and transcription factor occupancy on the indicated glycolytic gene promoters in Ishikawa cells. The graph shows the enrichment of genes relative to input. All data shown are mean ± SD of triplicate measurements that have been repeated three times with similar results.

c-Myc is a transcription factor that plays a critical role in regulating cellular metabolism and promoting tumor growth. It is one of the most commonly deregulated oncogenes in human cancers, and its overexpression or constitutive activation is observed in a wide range of cancer types. (21–23). Therefore, we speculate that AIB1 may induce the glucose metabolism reprogramming by c-myc in the endometrial cancer. In order to further explore the mode and extent of action of AIB1 affecting tumor glycolytic metabolism, we tried to compare the expression of transcription factor c-myc in the transcriptional co-activation complex after overexpression of AIB1 alone versus co-transfection of AIB1 and PCAF plasmid. Fortunately, the results showed that the ability of the transcriptional co-activation complex to bind transcription factors was significantly stronger than the recruitment of AIB1 alone (**Figure 6A**).

To next investigate how the transcription activation complex binds to the glycolytic enzymes promoter, which spans −2,000 to −1 (the translation initial site is 0), we performed chromatin immunoprecipitation (ChIP) and qPCR as shown in **Figure 6B**. The results showed that PCAF/AIB1/c-Myc could pull down the DNA fragment of the PFKL, ENO1, LDHA, PKM2, and GLUT1 promoter region but not PGK1 and HK2 (**Figure 6C**).

### 3.7 AIB1/glycolysis axis regulates endometrial cancer growth *in vivo*

**Figure 7:**
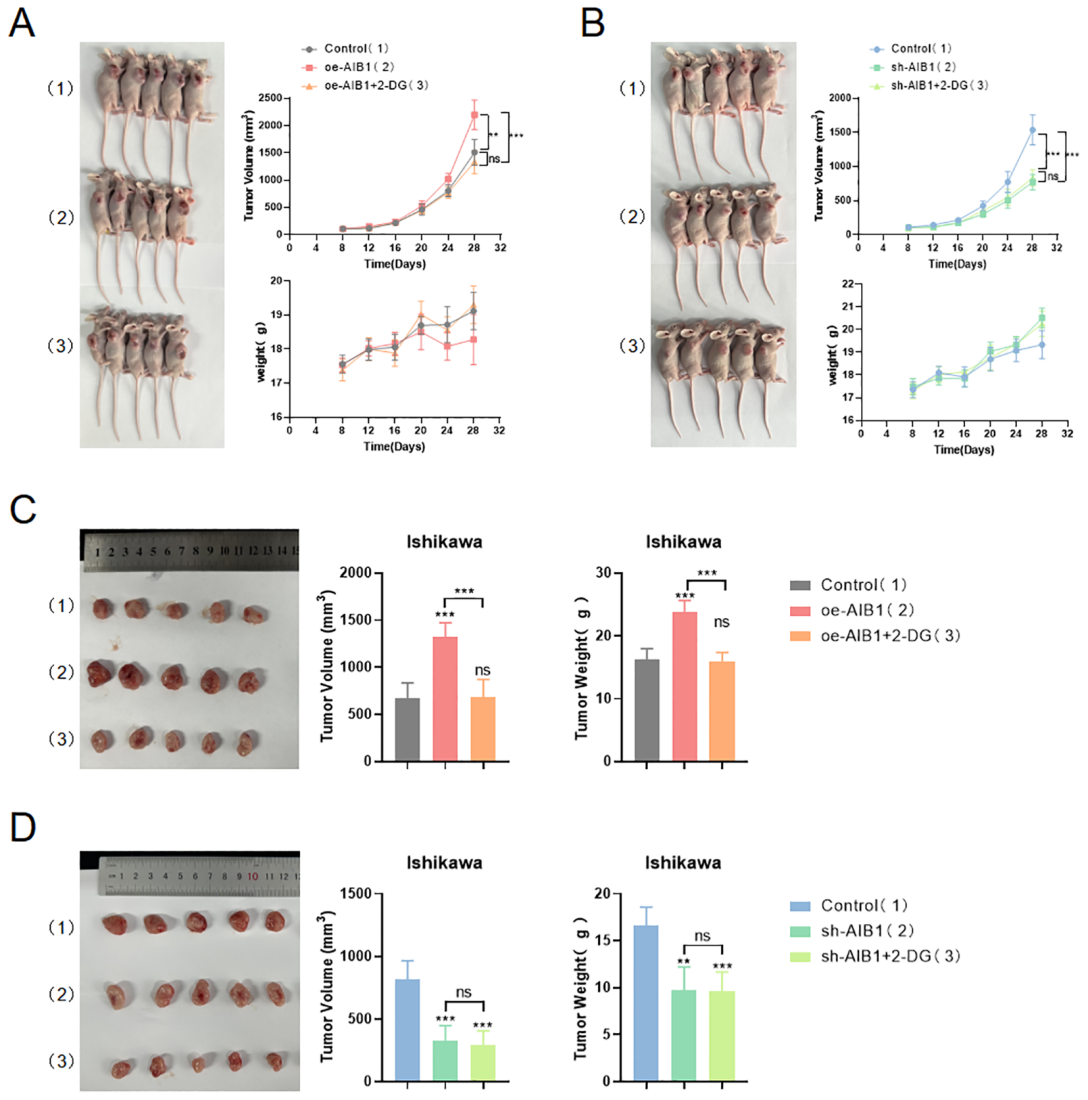
AIB1 regulates glycolysis and promotes tumor growth *in vivo.* Mice were randomly assigned to six groups (n = 5 per group) and were injected with vehicle (Control) or overexpression and knockdown AIB1 Ishikawa cells. Then immediately afterward, nude mice were treated with deoxy-d-glucose (2-DG) or equal volume of PBS. The effects of the drug on mouse weight and body tumor measurements are shown by the growth curve **(A, B)**. In addition, the excised tumors, tumor size, and tumor weight are shown **(C, D)**. Data are presented as the means ± SEM. ns, No significance.

On the basis of AIB1 regulating tumor proliferation and invasion in EC cells *in vitro*, we constructed the *in vivo* phenotype of the AIB1/glycolysis axis to perform subsequent verification. We examined the effect of the axis on tumor growth by subcutaneously injecting EC cells carrying the construct described in **Figure 7** into BALB/c nude mice. There was no significant difference in initial mouse body weight among groups. After the mice developed palpable tumors, they were randomly assigned into 2-DG or PBS in nude mice injected with Ishikawa cells overexpressing or knocking down the AIB1 gene. Unsurprisingly, overexpression of AIB1 significantly promoted endometrial tumor growth. Moreover, the tumor-promoting effect of AIB1 was significantly attenuated after pharmacological intervention with the glycolysis inhibitor 2-DG (**Figures 7A, C**). In contrast, when AIB1 was knocked down, tumor growth was inhibited and the effect of 2-DG intervention on tumor growth was not significant (**Figures 7B, D**).

## 4 Discussion

Endometrial carcinoma (EC) is a highly heterogeneous disease, with diverse etiologies, pathogeneses, clinical features, and molecular characteristics. Recent advancements, spearheaded by the landmark Cancer Genome Atlas (TCGA) project, have ushered in a shift from traditional morphology-based classification to a more nuanced molecular taxonomy of endometrial cancer. This four-subtype system, defined by POLE mutations, microsatellite instability, copy number alterations, and copy-number high tumors, has important prognostic and therapeutic implications (24–27).

Although there is no precise and effective means to screen and prevent endometrial cancer, endometrial cancer research has transitioned into the molecular era, with a growing focus on understanding the underlying molecular mechanisms driving its development and progression. This shift has allowed for the identification of molecular markers with prognostic significance, providing insights into the heterogeneous nature of the disease and potential therapeutic targets.

The molecule AIB1 (amplified in breast cancer 1), also known as SRC-3 (steroid receptor coactivator 3), has emerged as a promising prognostic marker in endometrial cancer. The AIB1 gene is located on chromosome 20q12 and consists of multiple exons. Previous studies have shown that AIB1 plays a crucial role in hormone signaling and glucose metabolic pathways and is associated with tumor progression, metastasis, and resistance to hormone-based therapies (28, 29). Building upon this molecular framework, our study aimed to elucidate the role of the transcriptional coactivator AIB1 (also known as SRC-3) in endometrial cancer progression and its potential as a clinically relevant biomarker.

Consistent with findings from public databases, our analysis of 66 endometrial cancer patients demonstrated that high AIB1 expression was an independent predictor of poor clinical outcomes. Mechanistically, we found that AIB1 plays a crucial role in promoting aberrant tumor metabolism, specifically by enhancing glycolysis. The acetylation of AIB1 at k687 by the acetyltransferase PCAF forms a transcriptional activation complex that binds to c-Myc, a master regulator of the glycolytic program. This metabolic reprogramming was functionally validated through cell-based assays, wherein the inhibition of glycolysis significantly attenuated the proliferative and invasive capacities of endometrial cancer cells. Notably, knockdown of AIB1 diminished the effects of glycolysis inhibitors, underscoring the central role of this coactivator in regulating these metabolic pathways. In addition, in combination with immunoprecipitation experiments, it was verified that the formation of a transcriptional activation complex between AIB1 and PCAF activates the glycolytic process to a greater extent than AIB1 alone to affect tumor progression.

These findings suggest that AIB1 may serve as a promising prognostic biomarker and a potential therapeutic target in endometrial cancer. Pharmacological strategies aimed at disrupting the AIB1-driven glycolytic network could hold clinical promise in curtailing disease progression. Whereas our findings provide valuable mechanistic insights into the role of AIB1 in endometrial cancer metabolism and progression, the current study has several limitations that should be acknowledged. The interconnected nature of cancer metabolism involves complex crosstalk between various pathways and interactions with the tumor microenvironment, which were not fully explored in this study (30). The focus on AIB1 and glycolysis may overlook immune microenvironment changing with metabolic reprogramming in endometrial cancer (31, 32). Additionally, our analysis was based on a relatively small patient cohort, and broader validation in larger, more diverse patient populations will be necessary to firmly establish the clinical utility of AIB1 as a prognostic biomarker. Future research should investigate the broader metabolic dependencies and vulnerabilities associated with AIB1 overexpression, as well as evaluate potential combinatorial therapeutic strategies targeting these metabolic alterations.

Despite these limitations, our study lays an important foundation for further exploration of AIB1 as a therapeutic target in endometrial cancer. Key next steps should include the development of specific AIB1 inhibitors and the evaluation of their efficacy in preclinical models and clinical trials. Deeper mechanistic understanding of how AIB1 coordinates transcriptional regulation of glycolytic and other metabolic pathways may also uncover additional druggable vulnerabilities. Integrating AIB1 assessment with comprehensive molecular profiling of endometrial tumors could also help refine patient stratification and guide the selection of tailored treatment approaches. Ultimately, a multifaceted approach combining biomarker development, metabolic targeting, and personalized therapy will be crucial to improving clinical outcomes for endometrial cancer patients.

## Data Availability

The datasets presented in this study can be found in online repositories. The names of the repository/repositories and accession number(s) can be found in the article/**Supplementary Material**.
