# Supplementary material for: PCAF acetylates AIB1 to form a transcriptional coactivator complex to promote glycolysis in endometrial cancer
Source: Front Oncol. 2024 Sep 5;14:1442965. doi: 10.3389/fonc.2024.1442965 (PMC11410763; doi:10.3389/fonc.2024.1442965)
Supplement: Supplementary file 1 [file Table1.docx]

|  |  | F | R |
| --- | --- | --- | --- |
| si-PCAF | siPCAF-1 | CCGCAUCAACUAUUGGCAUTT | AUGCCAAUAGUUGAUGCGGTT |
|  | siPCAF-2 | GCGACAACUCCUGGAACAATT | UUGUUCCAGGAGUUGUCGCTT |
|  | siPCAF-3 | CCGUAUGUUCCCAUCUCAATT | UUGAGAUGGGAACAUACGGTT |
| si-AIB1 | siAIB1-1 | GCUGAUAUCUGCCAAUCUUTT | AAGAUUGGCAGAUAUCAGCTT |
|  | siAIB1-2 | CCAGCAGAAUAUCAUGAUUTT | AAUCAUGAUAUUCUGCUGGTT |
|  | siAIB1-3 | GCAGCAGCAGAUGCUUCAATT | UUGAAGCAUCUGCUGCUGCTT |
| si-NC | | UUCUCCGAACGUGUCACGUTT | ACGUGACACGUUCGGAGAATT |

Supplementary Table 1.

| Gene | F | R |
| --- | --- | --- |
| PCAF | CCTCTTCTGGACTTGAGGCA | TTGGTCTCTGGTCCAAGCAT |
| AIB1 | TCAAGTATGGGTGGGCCAAA | CGAAGAGGCAATGTGGGAAT |
| GAPDH | ATCATCAGCAATGCCTCCTG | AGGCAGGGATGATGTTCTGG |
| PFKL | GACCGGAACTATGGGACCAA | CGGTGCTCGAAATCAGTGTC |
| ENO1 | GTGCAGCAAACTTCAGGGAA | CAGGATGTTGGGAGCAAACC |
| PKM2 | AGGAAGGTCCTGGGAGAGAA | GTTGCACCGTCCAATCATCA |
| LDHA | CTGTCATGGGTGGGTCCTTG | TGGGTGCAGAGTCTTCAGAG |
| PGK1 | CACTGTGGCTTCTGGCATAC | ACAATCTGCTTAGCCCGAGT |
| GLUT1 | GTATCGTCAACACGGCCTTC | AGAAGGCCACAAAGCCAAAG |
| HK2 | AAGTCCAGTGGAGTGGAAGG | CTTCCATGTAGCAGGCGTTG |

Supplementary Table 2
